# Epithelial Bone Morphogenic Protein 2 and 4 Are Indispensable for Tooth Development

**DOI:** 10.3389/fphys.2021.660644

**Published:** 2021-08-16

**Authors:** Haibin Mu, Xin Liu, Shuoshuo Geng, Dian Su, Heran Chang, Lili Li, Han Jin, Xiumei Wang, Ying Li, Bin Zhang, Xiaohua Xie

**Affiliations:** ^1^Department of Stomatology, The Second Affiliated Hospital of Harbin Medical University, Harbin, China; ^2^Institute of Hard Tissue Development and Regeneration, The Second Affiliated Hospital of Harbin Medical University, Harbin, China; ^3^Department of Stomatology, The First Affiliated Hospital of Harbin Medical University, Harbin, China; ^4^Heilongjiang Academy of Medical Sciences, Harbin, China

**Keywords:** tooth root, bone morphogenic protein, short root anomaly (SRA), Hertwig’s Epithelial Root Sheath (HERS), epithelial-mesenchymal interaction

## Abstract

The *Bmp2* and *Bmp4* expressed in root mesenchyme were essential for the patterning and cellular differentiation of tooth root. The role of the epithelium-derived Bmps in tooth root development, however, had not been reported. In this study, we found that the double abrogation of *Bmp2* and *Bmp4* from mouse epithelium caused short root anomaly (SRA). The *K14-cre*;*Bmp2*^*f/f*^;*Bmp4*^*f/f*^ mice exhibited a persistent Hertwig’s Epithelial Root Sheath (HERS) with the reduced cell death, and the down-regulated BMP-Smad4 and Erk signaling pathways. Moreover, the *Shh* expression in the HERS, the Shh-Gli1 signaling, and *Nfic* expression in the root mesenchyme of the *K14-cre*;*Bmp2*^*f/f*^;*Bmp4*^*f/f*^ mice were also decreased, indicating a disrupted epithelium- mesenchyme interaction between HERS and root mesenchyme. Such disruption suppressed the *Osx* and *Dspp* expression in the root mesenchyme, indicating an impairment on the differentiation and maturation of root odontoblasts. The impaired differentiation and maturation of root odontoblasts could be rescued partially by transgenic *Dspp*. Therefore, although required in a low dosage and with a functional redundancy, the epithelial Bmp2 and Bmp4 were indispensable for the HERS degeneration, as well as the differentiation and maturation of root mesenchyme.

## Introduction

Although the mammalian tooth is putatively regarded as an intact organ to fulfill physiological functions, the enamel-covered tooth crown and the cementum-covered tooth root actually undergo separated developmental processes which are regulated by different genetic mechanisms (Steele-Perkins et al., 2003). The development of tooth crown is divided into laminar, bud, cap, and bell stages according to the morphology of the epithelial-derived enamel organ (Luder et al., 2015). In the tooth germs of bell stage, the enamel organ differentiates into outer enamel epithelium (OEE), satellite reticulum, stratum intermediate, and inner enamel epithelium (IEE) from the external to internal side. At the apical edge of the enamel organ, OEE and IEE meet together to form a bilayer epithelium which elongates into Hertwig’s Epithelial Root Sheath (HERS). HERS induces not only the apical dental mesenchymal cells into the root odontoblasts which secret root dentin (Ten Cate, 1996), but also the dental follicle cells into cementoblasts to produce cementum (Kim et al., 2013). Eventually, the HERS degenerates and disappears in the erupted tooth, instead of differentiating into the enamel-secreting ameloblasts as the IEE does in the crown (Huang et al., 2009). A number of studies demonstrated that both the formation and degeneration of HERS were key to the length, shape, and number of tooth root, as well as the cementum and periodontal ligament (Bosshardt et al., 2015).

The initiation of tooth root, namely, the formation of HERS, starts almost at the late morphogenesis of the tooth crown. However, the reciprocal interactions between dental epithelium and the underlying mesenchyme, which are essential for the development of tooth crown (Chai et al., 2006), are also required during the development of tooth root (Huang et al., 2010). The induction of root odontoblasts and cementoblasts by HERS requires the direct contacts of HERS to root mesenchyme and dental follicle cells (Xiong et al., 2013). A lot of growth factors, such as BMPs, TGFβ, and SHH, were secreted by HERS to activate the pivotal transcription factor, *Nuclear Factor I C* (*Nfic*), in the root mesenchyme (Lee et al., 2009). Although *Nfic* transcription can be detected in both the crown and root odontoblasts, the *Nfic* null mice only showed the short tooth roots without overt HERS defects (Park et al., 2007), which indicated that the genetic network in root development was different from that in tooth crown.

Taking the advantages of the genetic animal models, a number of growth factors, transcription factors, and signaling pathways have proven to be involved in root development (Ono et al., 2016). During tooth root development, *Bmp2,3,4*, and *7* were expressed only in the root mesenchyme or pre-odontoblasts, as opposed to HERS (Yamashiro et al., 2003). The BMP ligands emanated from root mesenchyme are believed to activate *Sonic Hedgehog* (*Shh)* expression in HERS through Msx2 or BMP/Smad4 signaling (Li et al., 2015). Then, Shh secreted from HERS activates the transcription of *Nfic* in root mesenchyme through Gli1 (Li et al., 2015). Further, NFIC activates *Osterix* (*OSX*) in the precursors of pre-odontoblasts to promote the differentiation by enhancing *Dentin sialophosphoprotein* (*Dspp*) and *Dental Matrix Protein 1* (*Dmp1*) expression (Zhang et al., 2015). Inactivation of *Smad4* in mouse ectoderm and abrogating *Bmp Receptor IA* (*Bmpr1a*) by inducible *K14-cre* resulted in the complete loss of root and the short root anomaly (SRA), respectively (Huang et al., 2010), indicating that the BMP/Smad4 signaling in the ectoderm-derived HERS was essential for tooth root development.

Up to now, the transcription of *Bmp2,3,4*, and *7* has not been detected in HERS. However, when *Bmp2* and *Bmp4* were both inactivated by *K14-cre*, the mice exhibited not only the compromised amelogenesis (Xie et al., 2016), but also the shorter tooth roots. In this study, we generated *K14-cre;Bmp2^*f/f*^;Bmp4^*f/f*^* mouse to address if the BMP/Smad4 signaling in HERS is thoroughly contributed to by the mesenchymal BMP ligands, and the role of epithelial BMP ligands in tooth root development.

## Materials and Methods

### Mouse Lines

The *K14-cre* transgenic (Stock NO. 016230), *Bmp2*^*f/f*^ (Stock NO. 016878), and *Bmp4*^*f/f*^ (Stock NO. 018964) knock-in mice were obtained from Jackson Laboratory. The *Dspp* transgenic line was gifted by Dr. Chunlin Qin in Texas A&M College of Dentistry (Jani et al., 2016). To generate *K14-cre*;*Bmp2*^*f/f*^;*Bmp4*^*f/f*^ mice referred to as “*K14*-*Cre*-mediated double conditional knockout” (dcKO), the *Bmp2*^*f/f*^;*Bmp4*^*f/f*^ mice were crossbred with *K14-cre*;*Bmp2*^*f*/+^;*Bmp4*^*f*/+^ mice. To generate *K14-cre*;*Bmp2*^*f/f*^; *Bmp4*^*f/f*^;*Dspp*^*Tg*^ mice (referred to as dcKO;*Dspp*^*Tg*^), the *Bmp2*^*f/f*^;*Bmp4*^*f/f*^;*Dspp*^*Tg*^ mice were crossbred with *K14-cre*;*Bmp2*^*f*/+^;*Bmp4*^*f*/+^ mice. The genotyping procedures and primer sequences were described previously (Xie et al., 2016). All the mouse lines were bred and expanded in the Laboratory Animal Center at The Second Affiliated Hospital of Harbin Medical University. All the animal protocols (KY2016-087 and SYDW2019-2) were in accordance with the guidelines and approved by the research committee at The Second Affiliated Hospital of Harbin Medical University.

### Plain X-Ray Radiography and Micro-Computed Tomography

The mandibles dissected from the 3-week-old *Bmp2*^*f/f*^; *Bmp4*^*f/f*^(as normal control),*K14-cre*; *Bmp2*^*f*/+^;*Bmp4*^*f*/+^, *K14-cre*;*Bmp2*^*f/f*^;*Bmp4*^*f*/+^, *K14-cre*; *Bmp2*^*f*/+^;*Bmp4*^*f/f*^, and *K14-cre*; *Bmp2*^*f/f*^;*Bmp4*^*f/f*^ (dcKO) mice were fixed in 4% parafor- maldehyde (PFA) for 48 h at 4°C and then dehydrated to 70% ethanol gradually. Similarly, the mandibles from 3-week- old *Bmp2*^*f/f*^;*Bmp4*^*f/f*^;*Dspp*^*Tg*^ (as control), *K14-cre*;*Bmp2*^*f*/+^; *Bmp4*^*f*/+^;*Dspp*^*Tg*^, *K14-cre*; *Bmp2*^*f/f*^; *Bmp4*^*f*/+^;*Dspp*^*Tg*^, *K14-cre*;*Bmp2*^*f*/+^;*Bmp4*^*f/f*^;*Dspp*^*Tg*^, and *K14-cre*;*Bmp2*^*f/f*^;*Bmp4*^*f/f*^; *Dspp*^*Tg*^ (dcKO;*Dspp*^*Tg*^) mice were treated in the same procedures. Four pairs of jaws of each genotype (*n* = 4) were utilized for plain X-ray radiography by Faxitron MX-20 (Faxitron Bioptics, Tucson, AZ, United States). For micro-CT analysis, μCT35 (Scanco Medical, Brüttisellen, Switzerland) was applied for morphological observations with 3.5 μm slice increment. For mineral density and thickness of root dentin or cementum, 200 slices centered on the cut-through of the mesial root in the first molar were analyzed.

### Decalcified Paraffin Sections and Hematoxylin and Eosin (H&E) Staining

The mouse mandibles were dissected and fixed in 4% PFA and then decalcified in 15% ethylenediaminetetracetic acid (EDTA) solution at 4°C for 1–2 weeks. The mandibles were dehydrated with gradient alcohols and degreased with xylene for paraffin embedding. Serial sections were prepared in the thickness of 5 μm for hematoxylin and eosin (H&E) staining, PCNA assay, TUNEL assay, or immunohistochemistry (IHC) staining.

### Quantitative PCR

To evaluate the *Bmp2* and *Bmp4* abrogation in the dcKO HERS, the HERS was dissected from the P0 dcKO and WT first molars. Meanwhile, the oral mucosa of the P0 WT mice was also collected as negative control. The total RNA of the HERS and oral mucosa were extracted using RNeasy Kit (Qiagen) according to the manufacturer’s instructions. The complementary DNA (cDNA) was synthesized with the SuperScript^TM^ VILO^TM^ Master Mix (Invitrogen). The quantitative PCR was performed using SYBR Select Master Mix (Applied Biosystems, CA, United States) and the Quant Studio 6 Flux PCR System (Applied Biosystems). The *Bmp2* primers were 5′-GGGACCCGCTGTCTTCTAGT-3′ (forward) and 5′-TCAACTCAAATTCGCTGAGGAC-3′ (reverse); the Bmp4 primers were 5′-ATTCCTGGTAACCGAATGCTG-3′ (forward) and 5′-CCGGTCTCAGGTATCAAACTAGC-3′ (reverse).

### Cell Proliferation and Apoptosis Assay

The antibodies against PCNA (A0264, ABclonal) and Caspase 3 (96625, Cell signaling Technology) were applied to examine the cell proliferation and cell death in the molar roots. The paraffin sections were rehydrated with gradient alcohols after being de-paraffinized in xylene. Then, the sections were treated with 3% H_2_O_2_ and boiled citrate buffer for antigen retrieval. Prior to the overnight incubation with the antibodies against PCNA and Caspase 3, 3% bovine serum albumin was applied onto the section for 1 h in order to decrease the non-specific reactions. The combined secondary antibody and DAB kit (PV-6001, Zhongshan Golden Bridge Inc.) was used for the color development of the immuno-staining. Eventually, the sections were dehydrated with gradient alcohols and counter-stained with methyl green.

### Immunohistochemistry (IHC)

The decalcified paraffin sections for IHC eliminated the endogenous peroxidase activity with 3% H_2_O_2_ and retrieved antigens with boiled citrate buffer. Then, the sections were treated with 3% bovine serum albumin and 10% normal goat or rabbit serum to reduce non-specific immunoreactions. The sections were incubated with rabbit polyclonal primary antibodies against p-Smad1/5/8, p-Erk1/2, p-Junk, p-p38, DSP from Santa Cruz (Santa Cruz Biotechnology, Inc., Dallas, TX, United States), and rabbit polyclonal primary antibodies against Shh, Gli1, Nfic, OSX from Abcam (Abcam, Cambridge, MA, United States) overnight at 4°C and then the biotinylated-conjugated secondary antibodies (goat anti-rabbit antibodies) at room temperature for 1 h. The immunopositive loci were detected by the ABC kit and the DAB kit (Vector Laboratories, Burlingame, CA, United States) following the manufacturer’s instructions.

### *In situ* Hybridization (ISH)

The dissected mandibles were fixed in diethylpyrocarbonate (DEPC)-treated 4% PFA overnight and decalcified in DEPC-treated 15% EDTA solution at 4°C for 10 days. The mandibles were processed for paraffin serial section in the thickness of 10 μm. The DIG-labeled antisense RNA probe for mouse *Dentin sialophosphate protein* (*Dspp*) transcripts (Zhang et al., 2018) was prepared with DIG RNA labeling kit as per the manufacturer’s instruction (Roche, Indianapolis, IN, United States). An alkaline-conjugated antibody against DIG was used to detect RNA probes (Roche, Indianapolis, IN, United States) and the BM purple (VectorLaboratories, Burlingame, CA, United States) was used to develop positive signals. All the sections were counterstained with nuclear fast red. The detailed protocol of ISH was described previously (Zhang et al., 2018).

### Statistical Analyses

The measurements of the dentin thickness and the root length, the counting of the positive nuclear numbers in PCNA and Caspase 3, as well as the quantification of the immuno-staining intensity were all performed by Image J (version 1.46r, National Institutes of Health). The outcomes from Image J were statistically analyzed with student *T*-test by GraphPad Prism v8.2.1.441. All the statistical results were present in the mean with standard derivation (SD), which was regarded as significant only when the *p* value was less than 0.05.

## Results

### Double Abrogation of *Bmp2* and *Bmp4* in Ectoderm Led to SRA

**FIGURE 1:**
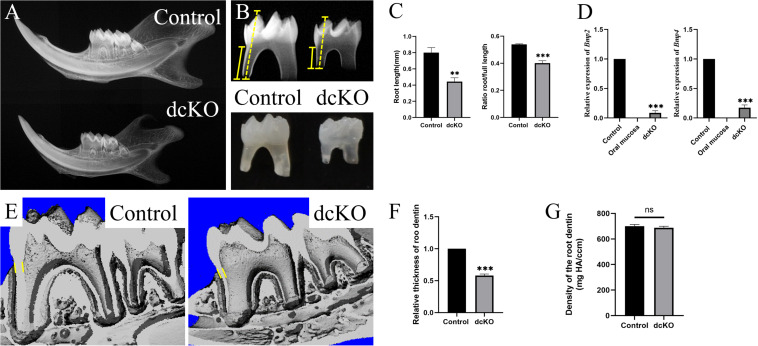
SRA in the dcKO molar. **(A)** The plain X-ray images of the P3W WT and dcKO mandibles. **(B)** The plain X-ray images of the P3W WT and dcKO first molars. **(C)** The statistical analysis of the root lengths of the P3W WT (0.802 ± 0.049 cm) and dcKO first molars (0.443 ± 0.038 cm; *p* = 0.0012). The ratios of root length to full length of the P3W WT (0.539 ± 0.004) and dcKO first molars (0.400 ± 0.014; *p* = 0.0002). **(D)** The quantitative PCR of the P0 HERS from dcKO and WT first molars. Compared to the WT controls, the relative expression of *Bmp2* was 0.08605 ± 0.03078 (*p* < 0.0001), and *Bmp4* 0.17603 ± 0.03775 (*p* < 0.0001). The P0 WT oral mucosa was used as a negative control. **(E)** The micro-CT images of the P3W WT and dcKO first molars. **(F)** The statistical analysis of the relative thicknesses of the dcKO first molar roots (0.54 ± 0.021; *p* < 0.001) to that of P3W WT molar roots. **(G)** Micro-CT scanning showed the gradient density of the P3W WT (700.40 ± 11.67 mg/ccm) and dcKO first molars (688.38 ± 10.23 mg/ccm; *p* = 0.0012). (The yellow dashed lines in panel **B** indicated the height of molar, and the yellow solid lines for the root height; the yellow lines in panels **E**,**F** delineated the measuring thickness of roots; ^∗∗^*p* < 0.01; ^∗∗∗^*p* < 0.001; ^ns^*p* > 0.05).

Our previous study showed that, although suffering from a severe Amelogenesis Imperfecta, the *K14-cre;Bmp2^*f/f*^;Bmp4^*f/f*^* mice (dcKO) could survive after weaning (Xie et al., 2016). Further investigation showed that both the mandibular bone and the molars were smaller than those of the normal littermates (Figures 1A,B). Consistently, the dcKO molar roots were dramatically shorter than the control molar roots. However, the ratio of root length to full tooth length in the dcKO molars was also significantly reduced compared to that of control molars (Figure 1C). The relative expression of *Bmp2* and *Bmp4* in the newborn dcKO HERS was also remarkably decreased compared to the WT counterparts (Figure 1D). These results suggested that the shortened dcKO molar roots resulted primarily from the impaired root development and the abrogated *Bmp2* and *Bmp4* expression, instead of secondarily to the decreased molar size. Additionally, the thickness of the root dentin in the dcKO molars was also obviously reduced (Figures 1E,F), though the density of the root dentin seemed little impacted (Figure 1G). Moreover, the SRA in dcKO molars was not detected in the *K14-cre*;*Bmp2*^*f*/+^;*Bmp4*^*f*/+^, *K14-cre*;*Bmp2*^*f/f*^;*Bmp4*^*f*/+^, or *K14-cre*;*Bmp2*^*f*/+^;*Bmp4*^*f/f*^ molars (Supplementary Figures 1A,B). Thus, only the double inactivation of *Bmp2* and *Bmp4* alleles in the epithelium could cause SRA in mouse molars.

### Reduced Cell Death Resulted in the Persistent HERS in dcKO Molar

**FIGURE 2:**
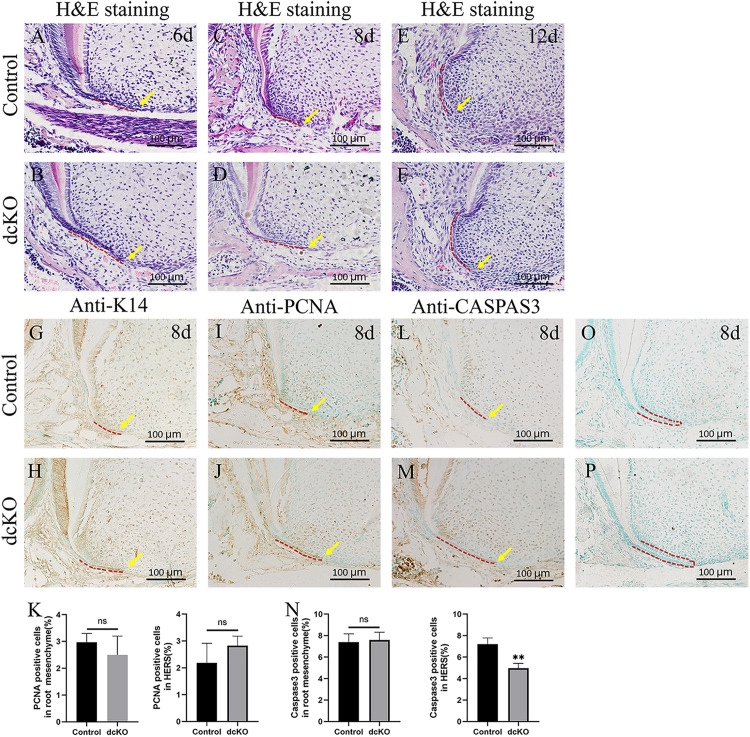
The cell proliferation and death in the persistent HERS of the dcKO molar root. **(A)** The H&E staining of the 1st molar root of P6 WT mouse. **(B)** The H&E staining of the 1st molar root of P6 dcKO mouse. **(C)** The H&E staining of the 1st molar root of P8 WT mouse. **(D)** The H&E staining of the 1st molar root of P8 dcKO mouse. **(E)** The H&E staining of the 1st molar root of P12 WT mouse. **(F)** The H&E staining of the 1st molar root of P12 dcKO mouse. **(G)** The K14 staining in the 1st molar root of P8 WT mouse. **(H)** The K14 staining in the 1st molar root of P8 dcKO mouse. **(I)** The PCNA staining in the 1st molar root of P8 WT mouse. **(J)** The PCNA staining in the 1st molar root of P8 dcKO mouse. **(K)** The statistical analysis of PCNA densities in the root mesenchyme and HERS. **(L)** The Caspase 3 staining in the 1st molar root of P8 WT mouse. **(M)** The Caspase 3 staining in the 1st molar root of P8 dcKO mouse. **(N)** The statistical analysis of Caspase 3 densities in the root mesenchyme and HERS. **(O)** Negative control for the anti-K14 immunostaining in the 1st molar root of P8 WT mouse. **(P)** Negative control for the anti-K14 immunostaining in the 1st molar root of P8 dcKO mouse. (The dashed red lines indicated the HERS; the yellow arrows delineated the end of HERS; ^∗∗^*p* < 0.01; ^ns^*p* > 0.05).

Since SRA was usually associated with the persistent HERS (Balic et al., 2019), a series of histological sections were prepared to examine the HERS morphology in the dcKO molars. H&E staining showed that at P6, when the control HERS of the 1st molar started to degenerate (Figure 2A), the dcKO HERS in the 1st molar stayed intact (Figure 2B). Such persistence of the dcKO HERS became more evident at P8 when the control HERS in the 1st molar exhibited an obvious degeneration (Figures 2C,D). At P12, there was almost no HERS detected in the control molar roots (Figure 2E), while the HERS in the dcKO 1st molar was still intact and kept the long-curved morphology (Figure 2F). To verify the persistence of the epithelium-derived HERS in the dcKO molars, the antibody against Keratin 14 was applied in the immunohistochemistry. The Keratin 14-positive cell band labeled an intact and long HERS in the dcKO 1st molar root even at P6 (Figure 2H), however, there was a short HERS with the Keratin 14 staining in the control molar roots at the same stage (Figure 2G). The persistent K14 positive cells could even be detected in the P10W periodontal ligaments (Supplementary Figure 2), confirming the persistent dcKO HERS. To explore how the HERS persisted, the cell proliferation and survival in the dcKO molar roots were examined. In the P8 1st molars, the PCNA densities in both the root mesenchyme and HERS showed no difference between the dcKO and control groups (Figures 2I–K). In contrast, although there was almost no difference in the root mesenchyme between the control and dcKO 1st molar, the Caspase3 assay displayed a decreased cell death in the HERS of the P8 dcKO 1st molar compared to the control counterpart (Figures 2L–N). Therefore, the persistent HERS in the dcKO molar most likely resulted from the decreased epithelial cell death, which was implicated to count for the SRA.

### Suppressed BMP Signaling Pathways in the HERS of dcKO Molar

**FIGURE 3:**
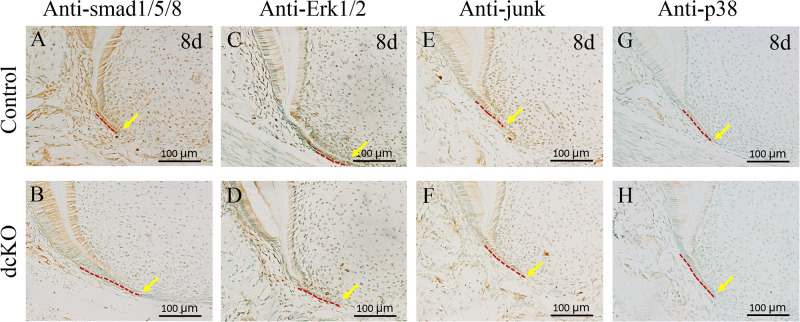
Smad-dependent and independent BMP signaling pathways in the dcKO molar root. **(A)** The immunohistochemistry with antibody against p-Smad1/5/8 in the 1st molar root of P8 WT mouse. **(B)** The immunohistochemistry with antibody against p-Smad1/5/8 in the 1st molar root of P8 dcKO mouse. **(C)** The immunohistochemical staining of p-Erk1/2 in the 1st molar root of P8 WT mouse. **(D)** The immunohistochemical staining of p-Erk1/2 in the 1st molar root of P8 dcKO mouse. **(E)** The p-Junk immunohistochemical staining in the 1st molar root of P8 WT mouse. **(F)** The p-Junk immunohistochemical staining in the 1st molar root of P8 dcKO mouse. **(G)** The immunohistochemistry with antibody against p-p38 in the 1st molar root of P8 WT mouse. **(H)** The immunohistochemistry with antibody against p-p38 in the 1st molar root of P8 dcKO mouse. (The dashed red lines indicated the HERS; the yellow arrows delineated the end of HERS).

To confirm the abrogation of *Bmp2* and *Bmp4* in the epithelium-derived HERS of dcKO molar, the Smad-dependent and independent BMP signaling pathways were checked. The intensity of p-Smad1/5/8 was significantly down-regulated in the persistent HERS of the P8 dcKO molar compared to the control molar HERS (Figures 3A,B). Moreover, the p-Smad1/5/8 intensity in the dcKO root mesenchyme was also reduced (Figures 3A,B), suggesting that the epithelial deletion of *Bmp2* and *Bmp4* also impacted root mesenchyme via epithelial-mesenchymal interactions. Similarly, both the p-Erk1/2 and p-Junk exhibited the decreased intensities in the HERS and root mesenchyme of the dcKO molars compared to the controls (Figures 3C–F). However, neither the intensity nor the distribution of the p-p38 in the dcKO molar differed from those in the control molars (Figures 3G,H).

### The Shh-Gli1-Nfic Interaction Was Disrupted in the dcKO Molar Root

**FIGURE 4:**
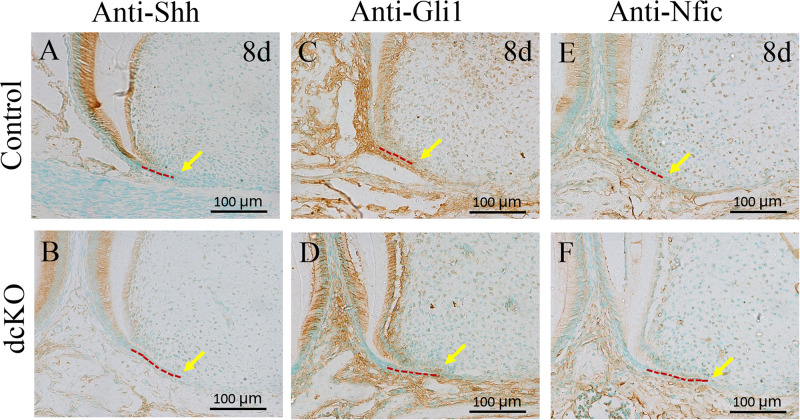
The immunohistochemical staining of the markers involved in tooth root development. **(A)** The Shh immunohistochemical staining in the 1st molar root of P8 WT mouse. **(B)** The Shh immunohistochemical staining in the 1st molar root of P8 dcKO mouse. **(C)** The immunohistochemical staining of Gli1 in the 1st molar root of P8 WT mouse. **(D)** immunohistochemical staining of Gli1 in the 1st molar root of P8 dcKO mouse. **(E)** The immunohistochemistry with antibody against Nfic in the 1st molar root of P8 WT mouse. **(F)** The immunohistochemistry with antibody against Nfic in the 1st molar root of P8 dcKO mouse. (The dashed red lines indicated the HERS; the yellow arrows delineated the end of HERS).

Previous studies demonstrated that the Bmp/Smad4 signaling in HERS controlled tooth root development by activating *Shh* expression in HERS (Huang et al., 2010), which then activated *Nfic* expression in the root mesenchyme (Liu et al., 2015). Compared to the control molar (Figure 4A), the *Shh* expression became obviously faint in the persistent HERS of the P8 dcKO molar (Figure 4B). Consistent with the reduced *Shh* expression, the numbers of the Gli1 positive cells were also reduced in both the P8 dcKO HERS and the root mesenchyme (Figures 4C,D). Similarly, as a down-stream target of Gli1, the expression of *Nfic* in the P8 dcKO molar was also detected in fewer mesenchymal cells compared to the control (Figures 4E,F).

### *Dspp* Transgene Partially Rescued the SRA in the dcKO Roots

**FIGURE 5:**
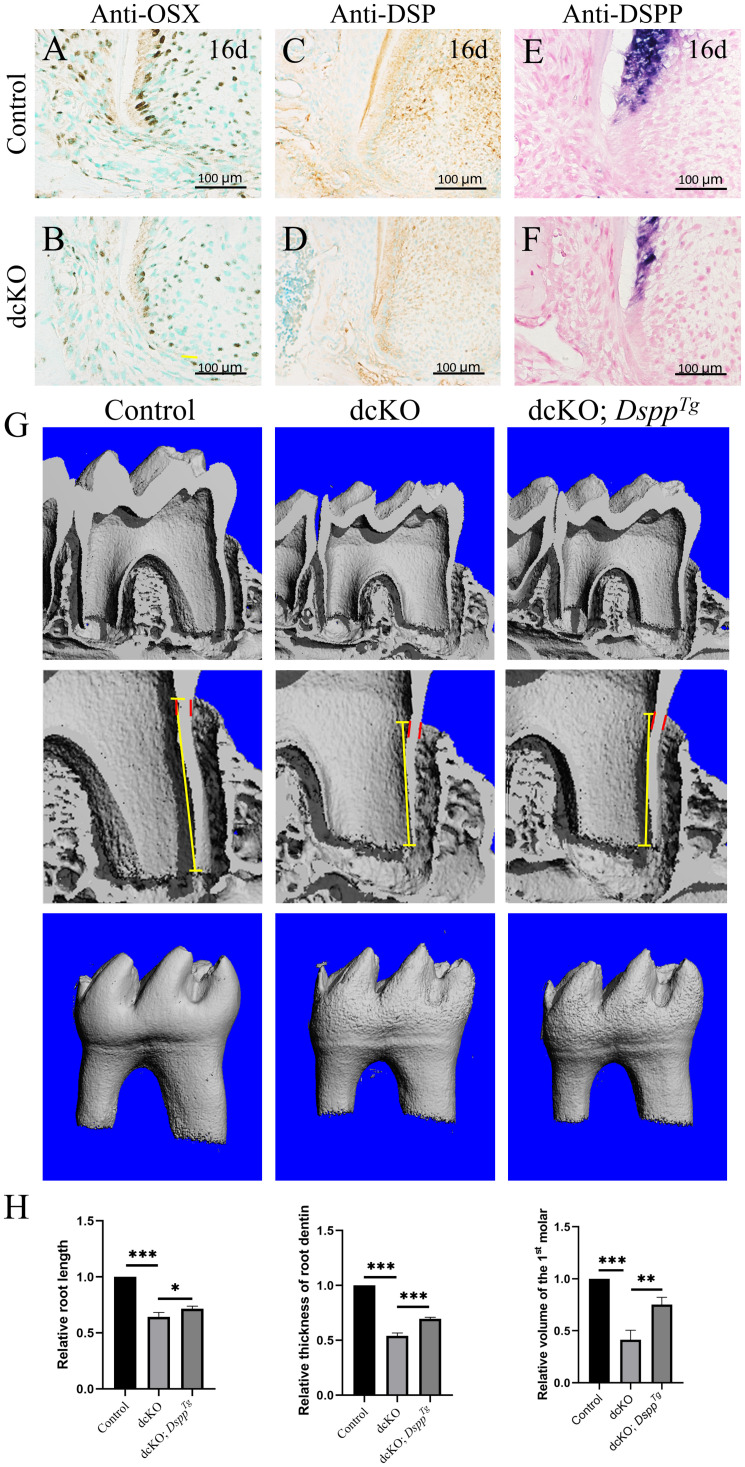
Expression of the odontogenic maturation markers and the morphology of the dcKO; *Dspp*^*Tg*^ molar root. **(A)** The immunohistochemistry with antibody against OSX in the 1st molar root of P16 WT mouse. **(B)** The immunohistochemistry with antibody against OSX in the 1st molar root of P16 dcKO mouse. **(C)** The immunohistochemistry staining of DSP in the 1st molar root of P16 WT mouse. **(D)** The immunohistochemistry staining of DSP in the 1st molar root of P16 dcKO mouse. **(E)** The *in situ* hybridization with *Dspp* anti-sense RNA probe in the 1st molar root of P16 WT mouse. **(F)** The *in situ* hybridization with *Dspp* anti-sense RNA probe in the 1st molar root of P16 dcKO mouse. **(G)** The micro-CT images of the molar root dentin in the P18 WT, dcKO, and dcKO; *Dspp*^*Tg*^ mice. **(H)** The statistical analysis of the root dentin length, thickness and volume in the P18 WT, dcKO, and dcKO; *Dspp*^*Tg*^ mice. (The dashed red lines indicated the HERS; the yellow arrows delineated the end of HERS; ^∗∗∗^*p* < 0.001; ^∗∗^*p* < 0.01; ^∗^*p* < 0.05).

As the pivotal transcription factor in tooth root development, Nfic played an essential role in the differentiation and maturation of root mesenchyme (Kim et al., 2015). To explore the odontogenic differentiation and maturation of the dcKO root mesenchyme, the expression of OSX and *Dspp* was examined. The immunohistochemical staining showed the OSX positive nucleus lining in the P16 control root odontoblast layer (Figure 5A), but only the sporadic OSX positive nucleus in the P16 dcKO root odontoblasts (Figure 5B). The DSP staining in the dcKO molar root was also much weaker than that of control molar (Figures 5C,D). The *in situ* hybridization with the *Dspp* anti-sense RNA probe further confirmed the reduced *Dspp* expression in the dcKO root odontoblasts (Figures 5E,F). To address if the SRA in the dcKO molars resulted from the impaired odontogenic differentiation and maturation, the molar roots of dcKO;*Dspp*^*Tg*^ mice were compared to those of control and dcKO mice. The micro-CT scanning showed that although still shorter and thinner than control root dentin, the root dentin of the P18 dcKO;*Dspp*^*Tg*^ molar was significantly longer, thicker, and larger than that of the dcKO molar (Figures 5G,H), indicating a partial rescue of the SRA in the dcKO molar by transgenic *Dspp*.

## Discussion

Short root anomaly is the most common phenotype in tooth root defects. In humans, the prevalence of SRA varies from 0.6 to 2.4% in different populations (Puranik et al., 2015). Genetically, the etiology of SRA could arrange from root mesenchyme to epithelial HERS (Huang et al., 2012). With regards to the role of BMP signaling in root development, previous studies demonstrated that *Bmps* were dominantly expressed in the root mesenchyme (Yamashiro et al., 2003), which was essential for the activation and maintenance of *Nfic* expression by activating Smad-dependent BMP signaling in HERS (Huang et al., 2010). However, up to now, there was no report on the involvement of HERS-derived BMPs in tooth root development. In this study, we reported that the deficiency of *Bmp2* and *Bmp4* in the epithelial HERS also resulted in SRA, which extends the insight of BMPs in tooth root development, as well as the etiological knowledge of SRA.

Different from the tooth root defects caused by the single deletion of *Bmp2* or *Bmp4* in the root mesenchyme (Torres et al., 2008; Guo et al., 2015), only the combined abrogation of *Bmp2* and *Bmp4* in ectoderm could result in SRA. The single deletion of *Bmp2* or *Bmp4* in ectoderm, or the double heterozygous of *Bmp2* and *Bmp4* deletion in ectoderm, or even the *K14-cre*;*Bmp2*^*f/f*^;*Bmp4*^*f*/+^, and *K14-cre*; *Bmp2*^*f*/+^;*Bmp4*^*f/f*^ mice exhibited normal tooth roots (Supplementary Figure 1). These results implicated that the ectoderm-derived Bmp2 and Bmp4 were not only required for the root development in a very low dosage, but also had a functional redundancy during root development.

During the morphogenesis of tooth root, HERS induced *Nfic* expression in root mesenchyme and then underwent degeneration (Lee et al., 2014). The delayed degeneration of HERS was usually associated with SRA (Huang et al., 2012). In the dcKO mice, the reduced cell death detected the persistent HERS, which was similar to the outcomes of the *Bmp2* or *Smad4* inactivation in root odontoblasts (Wang et al., 2012). The decreased cell death in the persistent dcKO HERS could be attributed to the ectodermal abrogation of *Bmp2* and *Bmp4*, thus, the epithelial *Bmp2* and *Bmp4* were suggested to promote the degeneration of HERS. However, it was hard to distinguish the ectoderm-derived or the mesenchyme-derived BMPs responsible for the delayed HRES degeneration because the BMP-Smad4 signaling was down-regulated in the HERS and root mesenchyme. A previous study demonstrated that the epithelial Smad4 was essential for the normal morphogenesis of HERS, as well as the root formation (Huang et al., 2010). Interestingly, the p-Erk1/2 and p-Junk were also reduced in the dcKO HERS, suggesting that the Erk and/or Junk signaling in HERS might be involved in the HERS degeneration. However, whether the epithelial BMP-Erk and/or BMP-Junk signaling play an essential role in the molar root development still requires further investigation, especially the phenotype of the *K14-cre;Erk2^*f/f*^* HERS and molar root.

In the dcKO molar roots, abrogation of *Bmp2* and *Bmp4* in ectoderm-derived HERS resulted in the reduction of BMP-Smad4 signaling in root mesenchyme, indicating a disruption of epithelium-mesenchyme interactions during root development. Since the BMP-Smad4 signaling in HERS was reported to activate *Shh* expression, which activated the *Nfic* expression in root mesenchyme (Li et al., 2015), the remarkable decrease of Shh in dcKO HERS and root mesenchyme, as well as the reduced Gli1 staining in dcKO root mesenchyme, were coincided to the down-regulated BMP-Smad4 signaling in the dcKO HERS. Consistent with the decreased Shh-Gli1 signaling in root mesenchyme, the Nfic immunohisto-staining in the dcKO root mesenchyme was found to be down-regulated, which indicated that the HERS-derived Bmps also was required for the differentiation and maturation of root mesenchyme.

*OSX* was regarded as a key down-stream target of Nfic because a series of conditional *OSX* knock-out mice exhibited a similar root phenotype to *Nfic* deficient mouse (Zhang et al., 2015). *OSX* was essential to activate *Dmp1* and *Dspp*, two key matrix proteins for root dentin (Zhang et al., 2018). Although the elongation and polarization of the root odontoblasts in the dcKO molars displayed no alteration, the reduced OSX-positive odontoblasts suggested an impaired odontogenic differentiation. The weakened DSP staining and reduced *Dspp* transcription also indicated a suppressed odontogenic differentiation and maturation of the root odontoblasts (Zhang et al., 2018). Therefore, the SRA in the dcKO mice was implicated in the suppression on root odontogenic differentiation. *Dspp* transgene at least partially rescued the significantly decreased length and thickness of the dcKO molar root, explicating that epithelial *Bmp2* and *Bmp4* deficiency impacted the root dentin formation through the epithelial-mesenchymal interactions.

In summary, our study indicated that although the ectoderm-derived Bmp2 and Bmp4 were not as robust as the Bmp2 and Bmp4 derived from root mesenchyme, they were critical to maintaining the epithelial-mesenchyme interaction during tooth root development, which was required for the degeneration of HERS, as well as the differentiation and maturation of root odontoblasts.

## Data Availability Statement

The original contributions presented in the study are included in the article/Supplementary Material, further inquiries can be directed to the corresponding authors.

## Ethics Statement

The animal study was reviewed and approved by the Ethics Committee at The Second Affiliated Hospital of Harbin Medical University. Written informed consent was obtained from the owners for the participation of their animals in this study.

## Author Contributions

XX and BZ contributed to the conception and design of the study. XL and SG wrote the initial draft of the manuscript. HM, DS, HC, LL, HJ, and YL performed the experimental work and data analysis. XW revised the manuscript. All authors have read and approved the final version of the manuscript.

## Conflict of Interest

The authors declare that the research was conducted in the absence of any commercial or financial relationships that could be construed as a potential conflict of interest.

## Publisher’s Note

All claims expressed in this article are solely those of the authors and do not necessarily represent those of their affiliated organizations, or those of the publisher, the editors and the reviewers. Any product that may be evaluated in this article, or claim that may be made by its manufacturer, is not guaranteed or endorsed by the publisher.

## Supplementary Material

Supplementary Figure 1 The plain X-ray images of the tooth root. **(A)** The plain X-ray images of the mandbiles from the P3W WT, *K14-cre; Bmp2^*f/*^*^+^*;Bmp4^*f/*^*^+^, *K14-cre; Bmp2^*f/f*^;Bmp4^*f/*^*^+^, *K14-cre; Bmp2^*f/*^*^+^*;Bmp4^*f/f*^*, and *K14-cre; Bmp2^*f/f*^;Bmp4^*f/f*^* (dcKO) mice. **(B)** The plain X-ray images of the first molars from the P3W WT, *K14-cre; Bmp2^*f/*^*^+^*;Bmp4^*f/*^*^+^, *K14-cre; Bmp2^*f/f*^;Bmp4^*f/*^*^+^, *K14-cre; Bmp2^*f/*^*^+^*;Bmp4^*f/f*^*, and *K14-cre; Bmp2^*f/f*^;Bmp4^*f/f*^* (dcKO) mice.

Supplementary Figure 2 The histology of the P10W dcKO 1st molar root. **(A)** The H&E staining of the 1st molar root of P10W WT mouse. **(B)** The H&E staining of the 1st molar root of P10W dcKO mouse. **(C)** The K14 staining in the 1st molar root of P10W WT mouse. **(D)** The K14 staining in the 1st molar root of P10W dcKO mouse. Arrows in panel **D** delineated the K14 positive cells.
