# Supplementary figures and images for: Epithelial Bone Morphogenic Protein 2 and 4 Are Indispensable for Tooth Development
Source: Front Physiol. 2021 Aug 16;12:660644. doi: 10.3389/fphys.2021.660644 (PMC8415269; doi:10.3389/fphys.2021.660644)

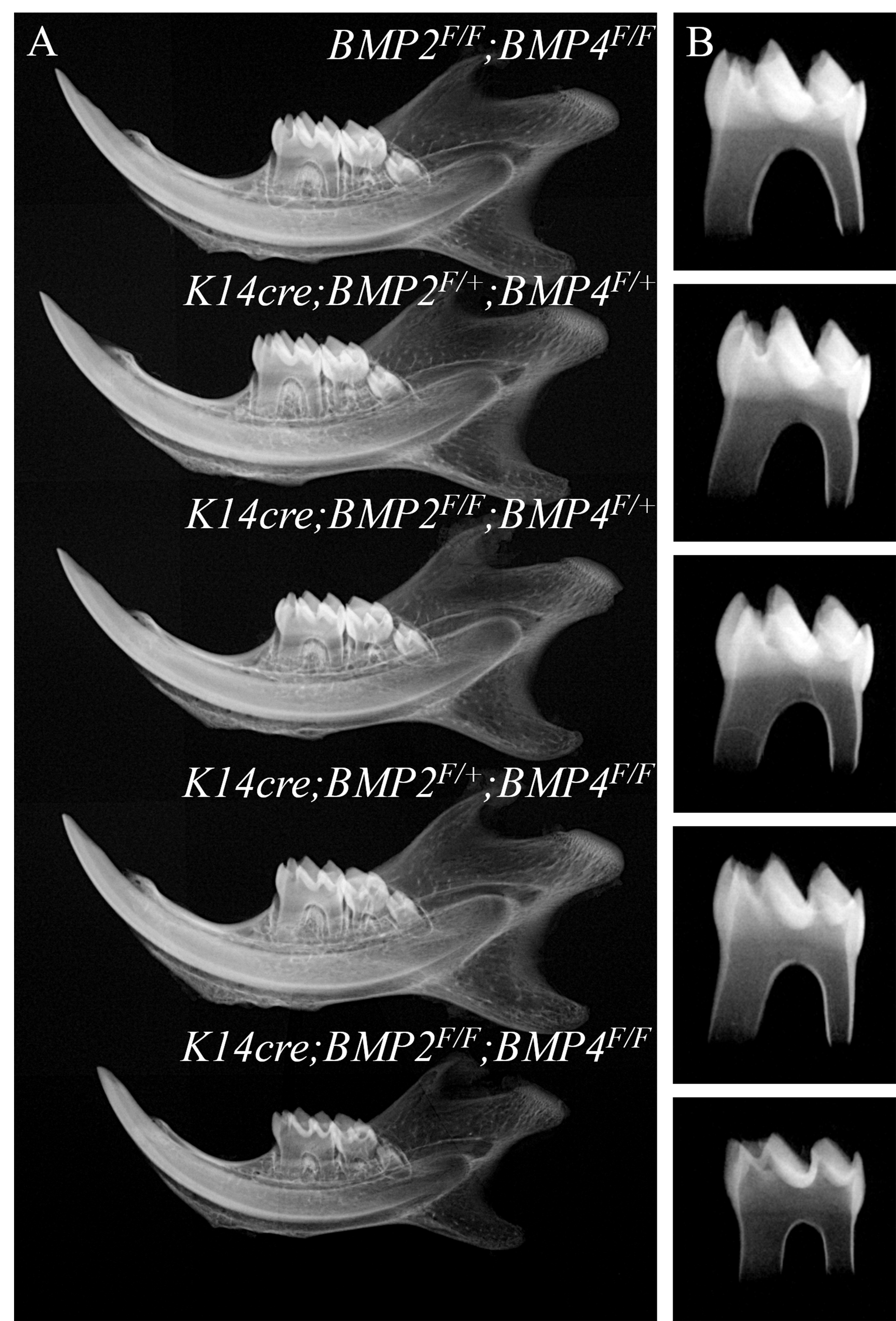

Supplement: Supplementary Figure 1 — The plain X-ray images of the tooth root. (A) The plain X-ray images of the mandbiles from the P3W WT, K14-cre; Bmp2f/+;Bmp4f/+, K14-cre; Bmp2f/f;Bmp4f/+, K14-cre; Bmp2f/+;Bmp4f/f, and K14-cre; Bmp2f/f;Bmp4f/f (dcKO) mice. (B) The plain X-ray images of the first molars from the P3W WT, K14-cre; Bmp2f/+;Bmp4f/+, K14-cre; Bmp2f/f;Bmp4f/+, K14-cre; Bmp2f/+;Bmp4f/f, and K14-cre; Bmp2f/f;Bmp4f/f (dcKO) mice. [file Image_1.TIF]

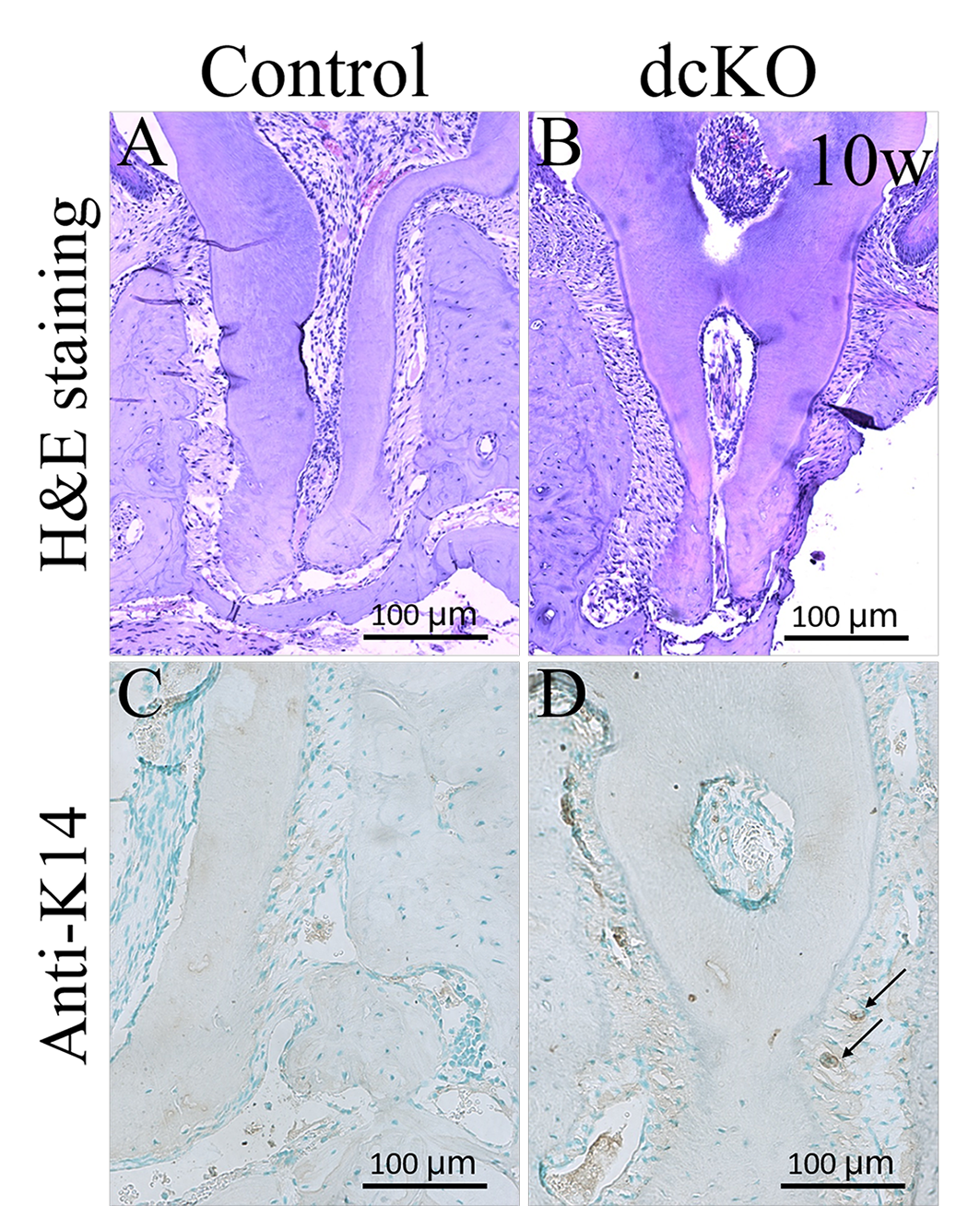

Supplement: Supplementary Figure 2 — The histology of the P10W dcKO 1st molar root. (A) The H&E staining of the 1st molar root of P10W WT mouse. (B) The H&E staining of the 1st molar root of P10W dcKO mouse. (C) The K14 staining in the 1st molar root of P10W WT mouse. (D) The K14 staining in the 1st molar root of P10W dcKO mouse. Arrows in panel D delineated the K14 positive cells. [file Image_2.TIF]
